# Preventing disease through opportunistic, *r*apid *e*ngag*em*ent by *p*rimary *c*are *t*eams using *b*ehaviour *c*hange *c*ounselling (PRE-EMPT): protocol for a general practice-based cluster randomised trial

**DOI:** 10.1186/1471-2296-11-69

**Published:** 2010-09-21

**Authors:** Clio Spanou, Sharon A Simpson, Kerry Hood, Adrian Edwards, David Cohen, Stephen Rollnick, Ben Carter, Jim McCambridge, Laurence Moore, Elizabeth Randell, Timothy Pickles, Christine Smith, Claire Lane, Fiona Wood, Hazel Thornton, Chris C Butler

**Affiliations:** 1 South East Wales Trials Unit, School of Medicine, Cardiff University, Neuadd Meirionnydd, Heath Park, Cardiff, CF14 4XN, UK; 2 Department of Primary Care and Public Health, School of Medicine, Cardiff University, Neuadd Meirionnydd, Heath Park, Cardiff, CF14 4XN, UK; 3 Health Economics and Policy Research Unit, Faculty of Health Sport and Science, University of Glamorgan, Pontypridd, CF37 1DL, UK; 4 Department of Public Health, Epidemiology & Biostatistics, University of Birmingham, Edgbaston, Birmingham, B15 2TT, UK; 5 Department of Social & Environmental Health Research, London School of Hygiene & Tropical Medicine, 15 - 17 Tavistock Place, London, WC1H 9SH, UK; 6 Cardiff Institute of Society and Health, School of Social Sciences, Cardiff University, 1-3 Museum Place, Cardiff, CF10 3BD, UK; 7 School of Nursing and Midwifery Studies, Cardiff University, Ty Dewi Sant, Heath Campus, Cardiff, CF14 4XN, UK; 8 School of Psychology, University of Birmingham, Edgbaston, Birmingham, B15 2TT, UK; 9 Department of Health Sciences, 2nd Floor Adrian Building, University of Leicester, Leicester LE1 7RH, UK

## Abstract

**Background:**

Smoking, excessive alcohol consumption, lack of exercise and an unhealthy diet are the key modifiable factors contributing to premature morbidity and mortality in the developed world. Brief interventions in health care consultations can be effective in changing single health behaviours. General Practice holds considerable potential for primary prevention through modifying patients' multiple risk behaviours, but feasible, acceptable and effective interventions are poorly developed, and uptake by practitioners is low. Through a process of theoretical development, modeling and exploratory trials, we have developed an intervention called Behaviour Change Counselling (BCC) derived from Motivational Interviewing (MI). This paper describes the protocol for an evaluation of a training intervention (the Talking Lifestyles Programme) which will enable practitioners to routinely use BCC during consultations for the above four risk behaviours.

**Methods/Design:**

This cluster randomised controlled efficacy trial (RCT) will evaluate the outcomes and costs of this training intervention for General Practitioners (GPs) and nurses. Training methods will include: a practice-based seminar, online self-directed learning, and reflecting on video recorded and simulated consultations. The intervention will be evaluated in 29 practices in Wales, UK; two clinicians will take part (one GP and one nurse) from each practice. In intervention practices both clinicians will receive training. The aim is to recruit 2000 patients into the study with an expected 30% drop out. The primary outcome will be the proportion of patients making changes in one or more of the four behaviours at three months. Results will be compared for patients seeing clinicians trained in BCC with patients seeing non-BCC trained clinicians. Economic and process evaluations will also be conducted.

**Discussion:**

Opportunistic engagement by health professionals potentially represents a cost effective medical intervention. This study integrates an existing, innovative intervention method with an innovative training model to enable clinicians to routinely use BCC, providing them with new tools to encourage and support people to make healthier choices. This trial will evaluate effectiveness in primary care and determine costs of the intervention.

**Trial Registration:**

ISRCTN22495456

## Background

Smoking, excessive alcohol consumption, lack of exercise and an unhealthy diet are the most important modifiable causes of premature morbidity and mortality in the developed world and may account for about 70% of health care expenditure [1]. Primary care in the UK is an important domain for preventive health care, since multiple, inter-related health behaviour problems are widespread and potentially amenable to rapid patient engagement and change. Over 80% of the population consult annually [2], and patients regard practitioners as a reliable source of advice. In primary care, people can receive help early on, when at lower risk. GPs and practice nurses also have the unique opportunity to intervene across a range of behaviours in the same individual, over time [3].

Efforts to change single risk behaviours using opportunistic brief interventions in health care consultations can be highly cost-effective [4,5] and have a small but important effect [6,7]. However, the *adoption *of these methods into routine care by primary care teams is sub-optimal [8]. There is a policy shift in the UK from, 'advice from on high to support from next door' [9].

Systematic reviews of primary prevention, through multiple risk factor engagement in primary care, conclude that existing evidence for effectiveness is inadequate [1,10]. Fleming and Godwin [11], the OXCHECK Study and British Family Heart studies [12,13] all showed modest effects. However, these studies relied on calling patients into the practice (i.e. the approach was not opportunistic), gave little attention to practitioner training or to individual patient plans, and the interventions were *not *based on motivational approaches that place the patient centre stage as the problem solver.

Over 20 systematic reviews of interventions to change practitioner behaviour [14,15] and reviews of those reviews [16,17] suggest that such interventions should be multifaceted, have a focused and active educational outreach component, include skills development, and be congruent with clinicians' values [18,19]. Nevertheless, there is as yet inadequate research on developing and evaluating interventions to facilitate discussion of health related behaviours in primary care consultations. There is an urgent need to test a generic intervention that enables practitioners to move routinely between multiple, inter-related risk behaviours, while respecting patients' inevitable motivational struggles [20,21].

### Motivational Interviewing and Behaviour Change Counselling

Motivational Interviewing (MI) is 'a facilitative, patient-centred counselling style for helping people explore and resolve ambivalence' by collaborating with them to articulate why and how they might change. Over the past 15 years, we have adapted MI, designed for use by specialists, into a generic, feasible and acceptable method to address the challenge of efficient, respectful and effective multiple-behaviour consultations in primary care [22,23]. Our method is called Behaviour Change Counselling (BCC) [24].

BCC incorporates a flexible menu-driven framework, with a definition and list of skills [24] designed specifically for brief health care consultations. It embraces the key elements of the '5 As' guideline (Assess, Advise, Agree, Assist, Arrange) [1], while also involving selection of concrete strategies according to individual need (e.g. establishing rapport, agenda setting, assessing importance and confidence, scaling questions, pros and cons, brainstorming solutions, negotiating attainable goals and follow-up).

A strong justification for an intervention like BCC is that unhealthy behaviours tend to be concentrated in the same people and strategies for addressing them may be better inter-linked [25]. Nevertheless, many patients with important risk factors do not receive interventions in primary care [8]. Reasons include: lack of time; lack of a sense of effectiveness; inadequate training and influences on clinician-patient relationships [26]. The 2003 GP contract in the UK encourages longer consultations (albeit only 10 minutes) and rewards effective health promotion, thus lowering one important barrier. However, implementation is unlikely to be increased by financial and organisational considerations alone. A generic, theory-driven method is needed that can be used by any professional group across behavioural domains which is sensitive to motivational struggles and does not leave patients feeling "preached to". It should also be satisfying for practitioners to use and for patients to receive.

The efficacy of methods such as brief intervention and cognitive-behavioural therapy depends on addressing two determinants of behaviour change embedded in prominent health psychology models: beliefs about the value of change (expectations of outcome or importance) and beliefs about one's capacity to succeed (self efficacy or confidence) [27-30]. Research on Motivational Interviewing (MI) [31] has introduced interpersonal predictors of behaviour change into this model by addressing the domains of importance and confidence within a relationship that can either hinder or promote motivation to change behaviour [32,33].

Literature [34-39] indicates that adaptations of MI are effective for a range of behaviour problems [40], superior to minimal or non-treatment controls and as good as, whilst being much briefer than, more intensive treatment interventions [41-44]. However, no study has evaluated training members of primary health care teams in BCC and examined efficacy on a range of patient behaviours.

Furthermore, simply advising practitioners to change their way of consulting is ineffective (e.g. antibiotic prescribing [45], brief alcohol intervention [46] and guidelines implementation [47]). Training is required to enhance practitioners' perceptions of the value of change and their ability to succeed [48]. This should ideally be an internally driven process [49,50], linked to everyday clinical challenges [23], adequately supported to ensure maintenance of change [51] and be properly evaluated [52].

We have developed two novel training methods [53,54] that are key to the "Talking Lifestyles" learning programme. They draw on the literature on clinician behaviour change and maximize the potential for acquiring and using new skills. The 'context-bound learning method' is a bottom-up, adult learning, experiential approach that relies on clinicians themselves evaluating the importance of the issue and then reflecting on authentic case scenarios. We have also developed a self-directed blended [55,56] e-learning program http://www.3trials.net that allows learners electronic access to video-rich clinical challenges before and after face-to-face training [57,58]. This method has potentially wide applicability at low cost and may assist in maintaining change.

### Research objectives

#### Primary Objective

The primary objective of the study is to evaluate the effect of training primary care health professionals in BCC on the proportion of patients self-reporting change in one or more of the four risk behaviours (smoking, alcohol use, exercise and healthy eating) at 3 months.

#### Secondary Objectives

The secondary objectives of this study are to evaluate the effect of training primary care health professionals in BCC on:

• Patient satisfaction, enablement and intention to change immediately post consultation.

• Patients' self-reported change at one year.

• Random cholesterol/HDL, salivary cotinine in individuals reporting quitting smoking, waist-to-hip ratio and blood pressure at one year.

• Practitioners' attitude and skills regarding opportunistic health promotion, and ease of adoption and implementation of skills gained in the training programme.

Economic costs, consequences and process evaluation of trial implementation analyses will be carried out including interviews with clinicians in both groups exploring their views of taking part in the trial.

The study has been approved by the Multi-centre Research ethics Committee (MREC, 07/MRE09/11) and all Local Health Boards (LHBs) in Wales.

## Methods/Design

### Study Design

This study is a cluster randomised controlled trial with randomisation at the level of practice. Behavioural outcomes will be assessed for eligible patients who consult with GPs and practice nurses exposed to training in BCC. These will be compared to outcomes for patients consulting in practices where GPs and practice nurses have not been exposed to training in BCC.

### Sample size

To show an increase in the proportion of patients reporting positive change on any one health behaviour from 50% to 65% at 3 months, an individually randomised study would require 340 patients. To account for clustering effects from randomised practices, with a moderate intra-cluster correlation (ICC) of 0.05, this is inflated to 1104 with 24 practices recruiting 46 patients each.

We initially planned to recruit 60 patients per practice, 30 during each recruitment week, 1440 in total, to allow for loss to follow-up of 30%. On average, each full time equivalent clinician will see between 20 and 40 patients a day, at least one quarter of whom are likely to be adults eligible for the study. This allows for considerable slippage and flexibility in meeting our target of 30 patients per practice per week for recruitment. However, following a poor 3 month follow-up response rate in the pilot study, we decided to recruit and randomise 29 practices (to allow for practice drop-out) and to continue recruitment beyond the 30 participants during each recruitment week. We implemented an early recruitment closure strategy in practices where the number of participants enrolled reached 40 and stopped recruitment at an appropriate time [59]. In addition to increasing the recruitment target, the approach and follow-up procedure was optimized based on recent evidence [60].

### Participants

#### General Practices

Twenty-nine general practices will be recruited, randomised and trained over 15 months. Each participating practice should have one GP and one nurse able to participate in the study for the duration of the intervention and evaluation and have adequate internet links (for accessing the training).

#### Patients

The patient inclusion criteria are: ability to provide informed consent, aged 18 years and over, attending in general practice, and screening above a designated risk threshold on any of the four behaviours studied. There are no specific exclusion criteria, other than that the patient must be able to understand and comply with the study protocol. We will therefore exclude people who are unable to complete the questionnaires in English. While we do not wish to exclude patients who might benefit from the intervention offered, in some cases, after a brief discussion, the clinician and the patient might decide that it would not be beneficial for the patient to participate in the study.

### Randomisation

Randomisation is undertaken using an optimal allocation approach [61]. Randomisation occurrs after all practices to be randomised within that block have consented, thereby ensuring allocation concealment. Once a block of practices has consented then all potential allocations to two groups are generated and a balance statistic is calculated based on practice list size and the modified Townsend deprivation score [62]. Those allocations that show the greatest degree of balance (1% with the smallest imbalance) are then passed blind to an independent statistician to randomly select an allocation in addition to randomly allocating group to intervention or control. Blocks of practices are being randomised in this way rather than all practices to allow for some practices to take longer to set up than others. Subsequent blocks will incorporate the degree of imbalance from previous ones to maintain balance across the study [63].

### Study Intervention

The *intervention *group will receive the blended BCC Training Programme; the *control *group will provide usual care with the offer of training after the study period. This course will move through clinician engagement, preparation, practice and maintenance, in which e-learning will be blended with face-to-face training, and ongoing support provided by MI trained facilitators. A pilot with two practices was used to construct the cases for the video-rich e-learning programme.

The intervention will be called the *Talking Lifestyle *learning programme, which takes practitioners through a portfolio-driven set of learning activities. Its goal is not to ensure complete clinical competence in the use of a guiding style for talking about behaviour change, but rather to start this process. As such it is an introduction to a set of skills that learners can practice and improve as they refine their efforts in everyday practice.

The programme derives much of its content from MI, but also integrates experience from other sources, like behaviour therapy. In developing the intervention the goal was to adapt these more complex psychological methods into a form that is applicable in the everyday practice of doctors and nurses in primary care. The intervention seeks to engage practitioners in thinking about the value of a more flexible shifting between communication styles with patients and to consider the more refined use of a guiding style when talking about behaviour change [24]. Of potential significance is the decision taken for practical reasons not to train clinicians in listening skills, which lie at the heart of motivational interviewing, but to focus on the more general adoption of a guiding style characteristic of BCC.

#### Structure

The structure of the course embraces a variety of learning opportunities, in this sequence:

• Part 1 - Seminar/Meet with the facilitator: Programme Induction

Individual participants will receive a personal/face-to face 1-hour programme induction from a facilitator trained in BCC.

• Part 2 - E-learning: Introduction

This part of the programme will orientate participants to the programme, the evidence pages, and judgments/views on behaviour change.

• Parts 3 and Part 4 - E-learning: Talking about behaviour change

Participants will be introduced to the core concepts of BCC and the concept of Flexible Consulting.

• Part 5 - Seminar/Meet the facilitator: Skills and strategies in practice

Participants will discuss the learning process/progress so far and prepare for the forthcoming simulated consultation.

• Part 6 - Practice/Simulated consultation: Feedback from facilitator

In this part of the programme, the participant conducts a simulated consultation with an actor/patient during a normal surgery session. The audio dialogue will be recorded, transcribed and reviewed by the facilitator, who will subsequently provide feedback to the participant possibly via telephone and/or a follow-up email.

• Part 7 - E-learning: Reflecting on practice by portfolio

Clinicians will be invited to undertake structured reflection and to report on the use of the consulting strategies during real consultations. This seeks to deepen the learning gained from the theoretical and hypothetical earlier stages [63].

• Part 8 - E-learning: Ongoing use of the PreEmpt ("Talking Lifestyle") network forum

A web forum will be accessible where clinicians can record and share experiences/reflections and educators can respond to clinicians' queries.

At 6 months a further simulated consultation with an actor/patient during a normal surgery session will be undertaken. The audio dialogue will be recorded, transcribed and reviewed by the facilitator, who will provide feedback to the participant. We intend that this will reinforce the learning from earlier stages of the programme. Throughout this whole period of study participation, clinicians can get in touch with facilitators by email or telephone and facilitators will be in touch, as required, to ask if they would like further support or advice.

### Outcome Measures and Tools

At 3 and 12 months, outcomes will be measured using four behaviour specific questionnaires. These are:

- Dietary Instrument for Nutrition Evaluation (DINE), evaluated in a UK population and measuring fat and fibre intake [64] and also the two item fruit and vegetable questionnaire [65];

- International Physical Activity Questionnaire (IPAQ). We will use the short form, assessing the last 7 days in self-administered format, a tool for measuring changes in overall physical activity [66];

- the Heaviness of Smoking Index [67] and

- the Alcohol Use Disorders Identification Test (AUDIT) and AUDIT-C questionnaire [68], both of which have been compared to biological markers.

Furthermore, there will be one question each assessing quality of life and general health [69,70]. At 12 months we will assess self efficacy for health behavior change using the Perceived Health Competence Scale (PHCS) [71]. The combined instrument questionnaire takes less than 20 minutes to complete.

The primary analysis will be based on the proportion of patients who show a clinically important positive change on any of these outcomes. The thresholds for positive change are defined as:

- a 20% reduction in alcohol consumption score,

- a 20% reduction in smoking scores,

- a 20% increase in healthy eating scores,

- a 20% increase in total physical activity scores.

Sensitivity analyses will explore the impact of both the magnitude of change considered important and any congruent negative changes which may affect conclusions of efficacy.

At the one year follow-up waist to hip ratio and Body Mass Index will be calculated, blood pressure will be measured and cholesterol/HDL test will be done using finger prick blood test. Salivary cotinine will be measured using a device called SmokeScreen [72]. While misreporting of smoking quit rates generally does not exceed 5%, data from this small sample will allow us to model possible effects in our study population [73].

### Trial procedure

#### Recruitment

##### Practice recruitment

We will write to practices explaining the study and inviting participation. The research team will telephone those not replying. The trial team will visit interested practices and provide additional information.

##### Patient recruitment

After training clinicians from intervention practices in BCC, both intervention and control practices will engage in two intensive, one-week periods of patient recruitment. The first one-week period will be within one month of training intervention clinicians in BCC. Five to seven months later, and after the second simulated consultation element of the BCC training for intervention practices, a second, similar period of recruitment will occur in each practice (intervention and control).

Patients seeing a clinician taking part in the study will be given a flyer (brief study information sheet) by the practice receptionist. After reading this sheet, patients interested in the study will be invited to approach a researcher who will be at the practice during the recruitment week. Interested patients will be given the complete patient information sheet and the consent form (full study documents pack). If they consent to participate, patients will be asked to fill out a questionnaire that includes tools for assessing smoking [55], risky drinking [56], physical activity [57], and dietary behaviour [70]. A screening form will be filled out by the researcher for each patient indicating if they have reached a threshold score on any of the instruments that could trigger intervention in UK general practice.

Participating patients will be given the full study document pack and the scored screening form to take with them to the consultation with the participating clinician. If deemed appropriate by their clinician, they will have the opportunity to discuss further their participation in the study and confirm or alter their decision. The clinician will counter sign the consent form and continue with the consultation and provide behaviour change counselling if appropriate. Patients not considered eligible and/or appropriate for participation by their clinician will not be followed any further. The completed study documents thus far (i.e. initial consent form, baseline questionnaire and screening form) will be kept by the research team. Participating patients who screen above the risk threshold(s) will see the researcher to fill in the post consultation questionnaire. Those who have consented to the study but who score below the risk thresholds will be given a thank you letter.

To facilitate smooth running of the study and minimise disruption to the practices during the recruitment phase, each clinician will keep four consultation appointments per day free and will be reimbursed for doing so. Posters will be put up in waiting rooms for a few weeks before recruitment begins to inform patients about the study.

#### Follow-up

Recruited patients will be sent a further questionnaire 3 months after recruitment. In order to improve response rates [60], initial non-responders will be sent a further reminder and questionnaire 2 weeks later. If they still do not respond they will be contacted by telephone 4 weeks after the initial questionnaire was sent and invited to either return the questionnaire by post or to complete it over the phone. An unconditional £5 gift voucher will be sent with each questionnaire as a token of appreciation or "thank you" [60].

Participants will be sent a further questionnaire 12 months after recruitment. Shortly afterwards, patients will be invited, either by the research team or the participating practices directly, to attend a follow-up appointment at their practice at a mutually convenient time/date. The location of these appointments will depend on staff and participating practices' availability and resources.

A £10 voucher redeemable at larger shops will be given to patients at the time of this visit in recognition of any travel expenses incurred and the time dedicated to attend the appointment. The researchers and practice staff (if appropriate) will be fully trained in the use of the devices for measuring cholesterol/HDL, cotinine and blood pressure. The results will be fed back to both clinicians and patients.

Patients will also be asked to consent to having their GP medical records checked for cholesterol, blood pressure, height and weight, duration of the index consultation, number of other consultations about health behaviours in the past year (since entering study) and relevant medications prescribed (e.g. nicotine replacement therapy or alternatives such as Bupropion or varenicline for smoking cessation, orlistat/sibutramine (sibutramine was available during the early part of the study, but was discontinued from availability in UK in January 2010) for weight loss, alcohol reduction treatment etc). The study team and the participating practices (if appropriate) will be responsible for research-related follow up.

### Analysis

#### Main Analysis

The primary outcome is a composite measure of positive change across four domains. The main analysis will be by intention to treat and will compare the two groups on the proportion of patients reporting positive change in behaviour at three months. This analysis will use a three level (four level if the practitioner level is found to be significant) logistic regression model (fitted using MLwiN v 2.02) to account for clustering at the practice, practitioner and patient level. Sensitivity analysis will be undertaken including alternative methods to allow for non-response.

Secondary outcomes of intentions and actual reported change in each individual behaviour (smoking, drinking, eating and exercise), validated change in smoking and BMI will be assessed using similar three-level (or four-level) regression models. Levels of patient enablement and satisfaction post consultation will be compared using multi level linear regression. Scores for general health will be compared between the two groups using multi level linear regression, allowing for repeated measurement at 3 months and one year and controlling for baseline levels. The two groups will be compared on the level of re-consultation with the practice and for certain prescription items (see 'Follow-up') in the 12 months after the index consultation, and the association between contact with trained practitioners and positive behaviour change will be assessed. Furthermore, the degree to which the participating patients feel capable of effectively managing their health at 12 months after the index consultation will be examined.

### Process Evaluation

The process evaluation will allow us to explore the impact of different aspects of the intervention, and particular barriers or facilitators towards implementation of the intervention [74]. We will:

1. Examine participation in the seminars and use of software supported learning. We will map the clinicians' use of the system (how often they log in, which pages they use) in relation to the primary outcome.

2. Complete qualitative interviews with around 15 clinicians in the control group to explore their views regarding practical aspects of taking part in the study and also the impact of the screening process on their practice. Around 15 clinicians in the experimental group will also be asked about these issues; however, these interviews will also explore ongoing use of the website, the communication skills, and the acceptability and perceived usefulness of various components of the intervention. The interviews will be conducted over the telephone and they will be audio recorded. They will last approximately 20-40 minutes. The items on the interview schedule and the analysis for the experimental group will be guided by the work of Richard Grol on issues to be considered when trying to change clinical practice [75].

3. Examine data from the simulated consultations to explore the intervention clinicians' competence in BCC using the Behaviour Change Counselling Index (BECCI) [76].

If the trial does not show an effect, the process evaluation will be useful in exploring possible reasons for this.

#### Qualitative Analysis

We will employ standard thematic analysis techniques, where transcripts will be closely examined to identify themes and categories [77]. Codes will be applied to these broad themes which will then be broken down further into sub-codes. Agreement on concepts and coding will be sought between members of the research team in order to ensure reliability. A proportion of the data (30%) will be coded by two different team members to check on reliability of the coding scheme. The interviewing will be iterative; where new themes emerge we will incorporate them into the interviews. Interviews will continue until all the themes are saturated, previous experience suggests that this will be around 30 interviews [78,45,79]. Thematic analysis will be supported by the use of computer assisted qualitative analysis software (NVIVO).

### Health Economics

As the intervention may have different impacts on each of the four studied behaviours, the economics of Pre-Empt will take the form of a costs - consequences analysis where costs will be assessed against the full range of outcomes. While such analysis cannot produce definitive conclusions regarding cost effectiveness, it will identify the costs of achieving any effects. Costs of the intervention will include the cost of training, potentially longer consultations in intervention practices, subsequent re-consultations and use of relevant NHS resources such as prescribed drugs (e.g. orlistat for weight loss) and specialist services (e.g. smoking cessation clinics). Duration of consultation will be estimated using clinical software. All other resource use will be monitored prospectively. Training costs will include trainer and learner time - including time online - as well as materials, travel, etc. Since education represents a one-off cost which may produce a stream of benefits over time, the costs of training will be annuitized over an (initial) presumed life of 5 years. Resources will be valued using standard methods [80] and mean differential costs between groups will be estimated.

To account for clustering effects, a two-level model will be used to estimate mean total costs for intervention and control groups [81]. Given the nature of the study design and the elements that make up the cost variable, it is unlikely that the data would be highly positively skewed. However, in the case of significant departure from normality, a generalised linear mixed model, which allows for random centre or practice effects but accommodates positively skewed data, will be used [82]. Multiple regression models for cost data will be estimated using MLwiN v2.02 and STATA v10 software.

## Discussion

This trial will be the first to evaluate the effectiveness of training primary care health professionals in BCC in a primary care setting using a novel blended learning training programme. The goal is to engage practitioners so that they use BCC in their health behaviour consultations in every day primary care. Opportunistic engagement by health professionals potentially represents one of the most cost effective medical interventions. This study integrates an existing training model and method into a blended learning programme and support service to enable practitioners to routinely use BCC by providing them with new tools to encourage and support people to make healthier choices.

The findings of this study will advance current knowledge within the field of patient and clinician behaviour change as well as the way educational interventions can efficiently and effectively be delivered, and inform future research. Patients who receive this intervention have the potential for improved long term health as well as improved communication with their nurse or GP; patients are seen as active problem solvers and drivers of their own behaviour change plans.

If the study shows the intervention is effective, then the training programme could be disseminated across the NHS so that all primary care practitioners can gain skills in talking to people about changing health related behaviour and the broader public will have greater opportunity to benefit from this intervention. This could improve the health of the population and in the long-term lead to a reduction in the cost of treating chronic diseases associated with risky behaviours. However, if the study shows no effect, then the research focus needs re-considering and/or the intervention examined for potential modifications.

### Protecting against bias

Practices who volunteer for the study are likely to be motivated to have access to the intervention, so those who are allocated to the control group may be disappointed. In order to avoid differential dropout between the experimental and control groups, we will offer those practices in the control group the opportunity to complete the training programme after the one year study period. Careful characterisation of the participating practices, clinicians and patients will be undertaken to judge the external validity of the study findings.

## Competing interests

The authors declare that they have no competing interests.

## Authors' contributions

CS led the writing of this manuscript, contributed to the protocol and intervention development, managed the trial and led the intervention delivery. CCB is the Principal Investigator who conceived the study and led the study design and funding application. SAS contributed to the study design, funding application, study implementation and the intervention development. KH contributed to the study design, management and led the statistical component of the study. DC contributed to the study design and the led the health economics component. SR contributed to the study design and the intervention development and delivery. AE, JMC, LM, CSm, CL, FW, HT contributed to the study design and management. BC contributed to the study design and analysis plan. ER contributed to data collection and input. TP contributed to the statistical analysis. SAS, KH, AE, DC, SR, CCB contributed to writing the paper. BC, JMC, LM, ER, TP, CSm, CL, FW and HT commented on drafts of the paper. All authors contributed to, read and approved the final version of the manuscript.

## Pre-publication history

The pre-publication history for this paper can be accessed here:

http://www.biomedcentral.com/1471-2296/11/69/prepub
